# Impact of Muscle Quality on Muscle Strength and Physical Performance Beyond Muscle Mass or Diabetes Status

**DOI:** 10.1002/jcsm.13760

**Published:** 2025-03-04

**Authors:** Jung A Kim, Chol Shin, Inha Jung, So Young Park, Da Young Lee, Ji Hee Yu, Hyunjoo Cho, Seung Ku Lee, Kyoung Jin Kim, Eyun Song, Kyeong Jin Kim, Nam Hoon Kim, Hye Jin Yoo, Sin Gon Kim, Kyung Mook Choi, Nan Hee Kim, Ji A Seo

**Affiliations:** ^1^ Division of Endocrinology and Metabolism, Department of Internal Medicine Korea University Anam Hospital Seoul Republic of Korea; ^2^ Institute of Human Genomic Study, College of Medicine Korea University Seoul Republic of Korea; ^3^ Biomedical Research Center Korea University Ansan Hospital Ansan Republic of Korea; ^4^ Division of Endocrinology and Metabolism, Department of Internal Medicine Korea University Ansan Hospital Ansan Republic of Korea; ^5^ Division of Endocrinology and Metabolism, Department of Internal Medicine Korea University Guro Hospital Seoul Republic of Korea

**Keywords:** muscle quality, muscle strength, myosteatosis, physical performance

## Abstract

**Background:**

Muscle quality, represented by myosteatosis, is recognized as an important factor in sarcopenia. In this study, we aimed to determine the associations between myosteatosis, muscle strength and physical performance among the elderly South Korean population.

**Methods:**

We included 1440 participants (mean age 62.7 ± 6.2 years) from the Korean Genome and Epidemiology Study (KoGES). Based on the computed tomography attenuation of mid‐thigh imaging, the total muscle area (TMA), normal‐attenuation muscle area (NAMA), low‐attenuation muscle area (LAMA) and inter‐intramuscular adipose tissue (IMAT) and its indices were used to evaluate myosteatosis. Muscle strength was evaluated using hand grip strength, whereas physical performance was evaluated through 4‐m gait speed, a 30‐s sit‐to‐stand test and 2‐min walking test.

**Results:**

Of the 1440 patients, 51.5% were women, and 37.2% had diabetes. With aging, the LAMA index gradually increased, and the NAMA index gradually decreased in both men and women (*p* for trend < 0.001). The NAMA index was positively associated, whereas the LAMA and IMAT indices were negatively associated with muscle strength and physical performance after adjusting for age and sex. Higher tertiles of the NAMA index were consistently associated with improved physical performance across all appendicular skeletal muscle tertiles. The relationship between the NAMA index or LAMA index and muscle strength and physical performance did not differ according to diabetic status. Regular exercise was associated with a higher NAMA index and a lower LAMA index in the non‐diabetic group; however, no significant difference in muscle quality was observed in the diabetic group in relation to exercise.

**Conclusions:**

Reduced myosteatosis was positively associated with greater muscle strength and better physical performance in both men and women, regardless of muscle mass or diabetes status; improving myosteatosis may be a therapeutic target for the prevention of sarcopenia.

## 1 Introduction

Sarcopenia is the loss of muscle function with declining muscle mass, which can lead to cardiometabolic disease, stroke, cognitive impairment, mobility disorders, falls, fractures and frailty, thereby contributing to mortality [1]. Previously, the loss of muscle mass was considered to play a pivotal role in sarcopenia. Since the 2010 European Sarcopenia Guidelines included grip strength and walking speed as necessary elements in the diagnostic criteria, muscle strength and function have been incorporated into the requirements in addition to muscle mass [2]. Muscle function is currently considered a proxy for sarcopenia [1]. Age‐related changes in muscle strength and physical performance occur faster than a decrease in muscle mass [3], and muscle quality, represented by myosteatosis, is recognized as an important factor in sarcopenia as well as muscle mass.

Myosteatosis, the infiltration of ectopic fat into skeletal muscle, is related to insulin resistance and systemic inflammation. It impairs muscle function and exacerbates the metabolic and functional decline associated with aging [4, 5]. From a Framingham heart study, lower computed tomography (CT) attenuation of paraspinal muscle—a marker of myosteatosis—was associated with metabolic risk factors such as hyperglycaemia, dyslipidaemia and hypertension [6].

Low muscle mass and myosteatosis are associated with muscle dysfunction. The effect of myosteatosis on muscle function may be independent of muscle mass or synergistic [7]. A study with African Caribbeans found that increased thigh inter‐intramuscular adipose tissue (IMAT) was associated with weaker grip strength and worse chair rise time and gait speed [8]. In a large cohort of older Caucasian men, lower muscle mass and lower calf muscle density were independently linked to worse physical performance, with minimal additional contribution from body fat [9]. Such impairments in lower body function are concerning, as they negatively impact activities of daily living and cognitive function, and are strong predictors of mortality [10].

Previous studies have shown that older subjects with diabetes have significantly lower muscle strength, muscle quality and lower calf muscle density compared to their nondiabetic counterparts [11, 12]. Considering the skeletal muscle is a major site for insulin‐stimulated glucose disposal [5], the impact of myosteatosis on muscle function may vary depending on diabetes.

CT is the most used modality to evaluate the extent and characteristics of myosteatosis. Muscle attenuation on CT is correlated with the lipid contents of the muscle from biopsy, which is the gold standard for evaluating myosteatosis [13]. To estimate myosteatosis, the total muscle area (TMA) is subdivided into normal‐attenuation muscle area (NAMA) and low‐attenuation muscle area (LAMA), and IMAT is also used [13, 14]. Recently, the NAMA/TAMA index, calculated by dividing NAMA by the total abdominal muscle area using abdominal CT imaging, has been suggested to be a reliable indicator of good muscle quality and has shown favourable metabolic characteristics [15].

In this study, we aimed to examine the associations between myosteatosis parameters such as NAMA, LAMA and IMAT, along with their respective indices, hand grip strength (HGS) and physical performance. We further investigated whether these correlations were modulated by the presence of diabetes or the extent of muscle mass.

## 2 Methods

### 2.1 Data Source and Study Population

Participants were enrolled in the Korean Genome and Epidemiology Study (KoGES). KoGES is an ongoing population‐based study of Ansan and Ansung cohorts in South Korea to evaluate the prevalence and risk factors for chronic diseases among the general population; it was initiated in 2001 and followed biennially [16, 17]. We collected health examination data, socioeconomic and demographic information, smoking and alcohol consumption histories, anthropometric examination data, physical capability assessment data and questionnaires. In this study, we selected 3204 participants from 8th to 9th visit (2015–2018) in the Ansan cohort. After excluding patients without data on HGS, usual gait speed, 30‐s sit‐to‐stand test (STST) and 2‐min walking test (2 MWT) data (*n* = 945), those without dual‐energy X‐ray absorptiometry (DXA) data (*n* = 132), those without CT images or myosteatosis values (*n* = 617) and those with insufficient diabetes information (*n* = 70), a total of 1440 participants were analysed (Figure S1). Before participation, all participants in the KoGES provided written informed consent to use their data. This study was performed in accordance with the principles of the Declaration of Helsinki of the World Medical Association and was approved by the Institutional Review Board of Korea University Ansan Hospital.

### 2.2 Assessment of Muscle Mass, Muscle Strength and Physical Performance

DXA (LUNAR Prodigy Advance; GE Medical Systems, Monterrey, Mexico) was used to evaluate the body muscle mass and body fat. Appendicular skeletal muscle mass (ASM [kg]) was defined as the sum of the lean soft tissue masses of the four limbs. We used ASM divided by height [2] as the skeletal muscle mass index (SMI). Low muscle mass was defined as an SMI of < 7.0 kg/m^2^ for men and < 5.4 kg/m^2^ for women based on the cut‐off points suggested by the Consensus Report of the Asian Working Group [18]. The body fat percentage (%) was used to evaluate the extent of fat mass.

HGS was used to assess muscle strength. HGS was measured using a digital dynamometer (TKK‐5401; TAKEI Science Instruments Co. Ltd, Nigata, Japan). To measure HGS, the participants were instructed to stand up, stretch both arms to prevent flexion and grip twice with maximum strength. A higher value was recorded for HGS (kg). The cut‐off points for low muscle strength were defined as an HGS of 28 kg for men and 18 kg for women [18].

Gait speed, STST and 2MWT [19] were used to assess physical performance. To measure gait speed, the participants were instructed to walk 4 m at a comfortable pace, and their walking speed was recorded as the usual gait speed (m/s) (VEL). In addition, we documented the fastest gait speed (m/s) (VEL‐F). Low physical performance was defined as slow gait velocity, characterized by a 4‐m gait speed of less than 1.0 m/s, as specified by the Korean Working Group on Sarcopenia guidelines [20]. The STST is a procedure that counts the total sit‐stand‐sit cycles during 30 s [21]. The cut‐off points for low STST were 14 and 12 for men and women, respectively. The cut‐off for the 2 MWT was 65 steps. Sarcopenia was defined as low muscle mass with low muscle strength or physical performance. Severe sarcopenia was defined as low muscle mass in the presence of low muscle strength and low physical performance. Functional sarcopenia is defined as low muscle strength and physical performance without loss of muscle mass [20].

### 2.3 Assessment of Muscle Quality

**FIGURE 1:**
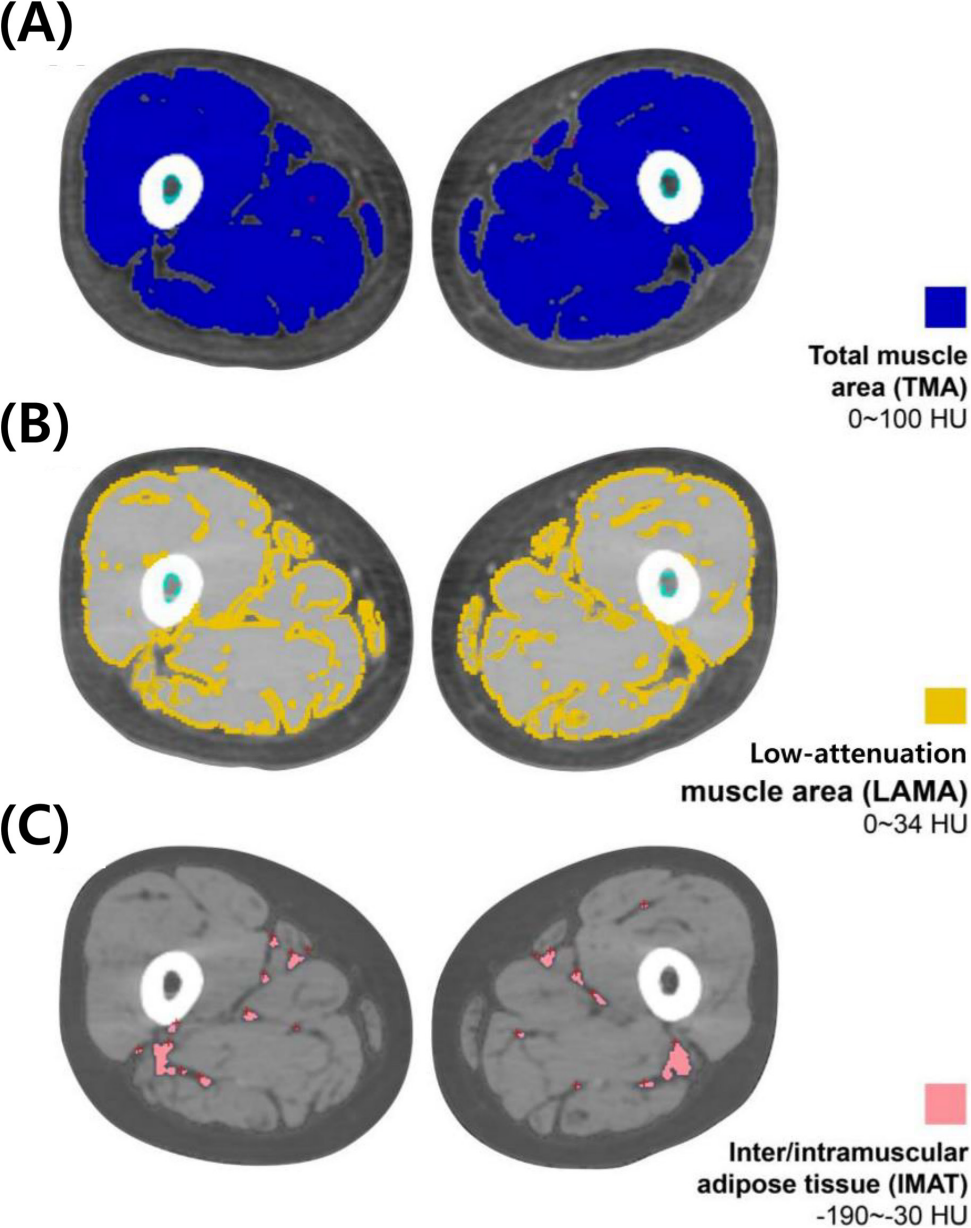
Computed tomography (CT) scan images of both mid‐thighs. (A) Blue: total muscle area (TMA): 0–100 HU, (B) yellow: low‐attenuation muscle area (LAMA): 0–34 HU and (C) red: inter/intramuscular adipose tissue area (IMAT): −190 to −30 HU. HU, Hounsfield unit.

Muscle quality, represented by myosteatosis, was measured using CT (Brilliance 64; Philips, Cleveland, OH, USA). A single‐slice transverse CT image at the midpoint between the upper border of the patella and the greater trochanter of the femur was used according to a standardized protocol. The two‐dimensional cross‐sectional area of the skeletal muscle at the thigh was analysed using Rapidia 3D software (v2.8; INFINITT Healthcare, Seoul, South Korea). The automated system measured the TMA and IMAT. The TMA was further divided into the LAMA and NAMA. The reference range of the Hounsfield units (HU) on CT is the following: TMA: 0–100 HU, NAMA: 34–100 HU, LAMA: 0–34 HU, IMAT: −190 to −30 HU [13, 14]. Additionally, we calculated the ratios of NAMA to TMA + IMAT (NAMA index), LAMA to TMA + IMAT (LAMA index) and IMAT to TMA + IMAT (IMAT index) (Figure 1) [15].

### 2.4 Definition of Covariates

After 8–12 h of overnight fasting, blood samples were collected and sent to Seoul Clinical Laboratory (Seoul, South Korea). To assess insulin resistance and beta cell function, homeostasis model assessment of insulin resistance (HOMA)‐IR and HOMA‐β were calculated, and the formulas are as follows: HOMA‐IR = [fasting insulin (μU/mL) × fasting plasma glucose (mg/dL)]/405; HOMA‐β = 20 × fasting insulin (μU/mL)/[fasting plasma glucose (mg/dL) − 63] [22]. Type 2 diabetes was defined as fasting plasma glucose ≥ 126 mg/dL or postprandial 2‐h glucose after ingestion of 75 g of glucose ≥ 200 mg/dL, or HbA1C ≥ 6.5%, or on anti‐diabetic medications [23]. Hypertension was defined as systolic blood pressure ≥ 140 or diastolic blood pressure ≥ 90 or on anti‐hypertensive medications [23]. Additionally, we examined several lifestyle determinants, including smoking status (non‐smoker, ex‐smoker, or current smoker) and alcohol consumption (g/week). Exercise was categorized as none, light or regular, with regular exercise defined as engaging in physical activity for more than 30 min at least three times per week [24]. Total metabolic equivalent of task (METs) was calculated using the formula: intensity of activities (MET) × duration (minutes) × frequency (days per week), to evaluate the impact of exercise type, intensity and frequency on muscle quality and function [25]. Educational background was also investigated and categorized into three groups: ≤ 6 years, 6–12 years or > 12 years.

### 2.5 Statistical Analysis

Data are represented as medians and interquartile ranges for non‐normally distributed variables and means ± standard deviations for normally distributed variables. Categorical variables are expressed as numbers and percentages. Continuous variables were compared using a one‐way analysis of variance (ANOVA). Variables including the NAMA, NAMA index, LAMA, LAMA index, IMAT, and IMAT index were log‐transformed to achieve normality before analysis. Categorical variables were analysed using the chi‐squared test. Age‐ and sex‐adjusted linear regression was used to evaluate correlations between muscle strength or physical performance and muscle mass or quality index. Multivariable linear regression was used to explore the relationship between muscle quality index and muscle strength or physical performance after adjusting for age, sex, ASM, exercise, alcohol consumption, smoking, cardiovascular disease, hypertension medication, lipid‐lowering medication and education. To examine the potential modifying effect of diabetes on these relationships, *p*‐values for interactions were calculated. To clarify the effect of regular exercise or total METs on muscle quality index, we performed age‐ and sex‐adjusted subgroup analyses based on diabetes status. Sensitivity analysis was conducted by excluding participants with a history of thyroid medication use (*n* = 42) and oestrogen use (*n* = 16). All statistical analyses were performed using SAS software (version 9.4, SAS Institute Inc., Cary, NC, USA), and a *p*‐value < 0.05 was considered statistically significant.

## 3 Results

### 3.1 Characteristics of the Study Population

Of the 1440 participants, 48.5% (*n* = 699) were men, and 51.5% (*n* = 741) were women (Table 1). The prevalence of diabetes was 39.8% (*n* = 278) and 34.6% (*n* = 257) in men and women, respectively. The diabetes group showed higher values for age, body mass index, waist circumference, fasting glucose, insulin, HOMA‐IR, systolic blood pressure, triglycerides and prevalence of hypertension. However, we observed no significant differences in smoking status between those with diabetes and those without. Women with diabetes showed higher total METs than women without. The ASM and SMI were higher in men without diabetes than in men with diabetes. On the contrary, women with diabetes had higher SMI than women without diabetes. After adjusting for age, the differences in ASM and SMI according to diabetes status in men were attenuated, whereas higher ASM and SMI were more clearly observed in women with diabetes (Table S1). Men in the non‐diabetes group had higher TMA, NAMA, NAMA index, HGS, VEL, VEL‐F, STST and 2MWT scores and lower LAMA, LAMA index, IMAT and IMAT index than those in the diabetes group. In women, the non‐diabetes group had higher NAMA index, STST and 2MWT and lower LAMA, LAMA index, IMAT and IMAT index than the diabetes group; however, we found no significant differences in TMA, NAMA, HGS, VEL and VEL‐F between the two groups. After adjusting for age, we found that NAMA and NAMA index, HGS, VEL, VEL‐F and 2MWT were lower, and LAMA and IMAT and their respective indices were higher in men with diabetes than in those without. However, no significant differences were noted between women with diabetes and those without, except for LAMA, IMAT and IMAT index (Table S1). Both men and women with diabetes showed a higher prevalence of low VEL and functional sarcopenia, whereas an increased proportion of patients with low HGS and severe sarcopenia was observed only in men with diabetes.

**TABLE 1:** Baseline characteristics.

	Men			Women		
	Non‐diabetes (*n* = 421)	Diabetes (*n* = 278)	*p*	Non‐diabetes (*n* = 484)	Diabetes (*n* = 257)	*p*
Age (years)	62.2 ± 5.8	64.4 ± 6.4	< 0.001	61.5 ± 5.6	63.7 ± 6.7	< 0.001
Body mass index (kg/m^2^)	24.4 ± 2.6	25.0 ± 2.8	0.002	24.2 ± 2.9	25.1 ± 3.3	< 0.001
Waist (cm)	85.4 ± 7.1	87.8 ± 7.9	< 0.001	79.9 ± 7.8	83.1 ± 8.2	< 0.001
Fasting glucose (mg/dL)	93.2 ± 8.0	117.9 ± 32.3	< 0.001	89.1 ± 7.4	112.8 ± 35.1	< 0.001
HbA1c (%)	5.5 ± 0.3	6.6 ± 1.2	< 0.001	5.6 ± 0.3	6.7 ± 1.4	< 0.001
Fasting insulin^a^	7.6 (5.8–9.8)	8.0 (6.2–10.8)	0.002	7.5 (6.0–1.0)	8.8 (6.8–11.4)	< 0.001
HOMA‐IR^a^	1.7 (1.3–2.2)	2.3 (1.7–3.1)	< 0.001	1.6 (1.3–2.2)	2.3 (1.7–3.3)	< 0.001
HOMA‐β	99.8 ± 40.9	75.5 ± 54.5	< 0.001	118.7 ± 48.8	89.2 ± 52.8	< 0.001
SBP (mmHg)	118.7 ± 13.3	120.2 ± 14.3	0.176	114.4 ± 15.7	118.9 ± 14.4	< 0.001
Total cholesterol (mg/dL)	190.0 ± 31.7	174.6 ± 34.4	< 0.001	206.2 ± 36.6	185.4 ± 37.8	< 0.001
HDL (mg/dL)	44.1 ± 10.7	41.8 ± 9.5	0.003	50.2 ± 11.8	46.9 ± 11.4	< 0.001
Triglyceride (mg/dL)^a^	119.3 (87.7–162.7)	134.6 (93.6–179.5)	0.092	108.4 (81.7–146.4)	123.2 (89.6–177.6)	< 0.001
ASM (kg)	22.8 ± 2.7	22.2 ± 2.7	0.012	15.3 ± 1.7	15.6 ± 1.8	0.062
ASM/height^2^	8.1 ± 0.8	8.0 ± 0.8	0.136	6.3 ± 0.6	6.5 ± 0.6	< 0.001
Body fat percent (%)	23.5 ± 5.5	24.8 ± 5.9	0.005	35.6 ± 5.3	35.2 ± 5.9	0.323
TMA (cm^3^)	136.8 ± 18.9	132.9 ± 18.0	0.006	91.9 ± 12.5	93.5 ± 13.3	0.111
NAMA (cm^3^)^a^	119.4 (108.8–132.0)	114.4 (100.8–126.8)	< 0.001	74.8 (66.6–83.0)	74.9 (64.9–84.6)	0.770
LAMA (cm^3^)^a^	16.0 (13.1–19.8)	18.1 (14.3–22.0)	< 0.001	16.1 (13.4–19.6)	17.4 (14.3–21.8)	0.001
IMAT (cm^3^)^a^	0.9 (0.6–1.4)	1.2 (0.8–1.8)	< 0.001	1.2 (0.7–1.7)	1.5 (1.0–2.1)	< 0.001
NAMA index^a^	87.8 (84.7–89.8)	85.6 (81.9–88.6)	< 0.001	81.4 (76.7–84.5)	79.8 (74.5–83.6)	0.001
LAMA index^a^	11.6 (9.7–14.3)	13.3 (10.8–16.6)	< 0.001	17.3 (14.3–21.5)	18.6 (15.1–23.1)	0.010
IMAT index^a^	0.7 (0.5–1.0)	0.9 (0.6–1.4)	< 0.001	1.3 (0.8–1.8)	1.6 (1.0–2.1)	< 0.001
HGS (kg)	36.3 ± 5.8	34.0 ± 6.2	< 0.001	21.4 ± 4.1	20.8 ± 4.0	0.071
VEL (m/s)	1.12 ± 0.19	1.06 ± 0.19	< 0.001	1.05 ± 0.16	1.03 ± 0.20	0.090
VEL‐F (m/s)	1.52 ± 0.26	1.44 ± 0.25	< 0.001	1.39 ± 0.20	1.37 ± 0.23	0.146
STST (counts)	19.2 ± 4.8	18.2 ± 4.5	0.004	17.2 ± 4.2	16.1 ± 4.1	0.002
2MWT (steps)	150.8 ± 28.1	135.3 ± 30.0	< 0.001	134.0 ± 27.5	127.7 ± 29.1	0.004
Low muscle mass (*n*, %)^a^	25 (5.9%)	24 (8.6%)	0.172	30 (6.2%)	8 (3.1%)	0.070
Low HGS (*n*, %)	27 (6.4%)	40 (14.4%)	0.001	93 (19.2%)	61 (23.7%)	0.149
Low VEL (*n*, %)	116 (27.6%)	114 (41.0%)	< 0.001	184 (38.0%)	124 (48.3%)	0.007
Low STST (*n*, %)	40 (9.5%)	38 (13.7%)	0.087	24 (5.0%)	26 (10.1%)	0.008
Low 2MWT (*n*, %)	1 (0.2%)	3 (1.1%)	0.149	1 (0.2%)	3 (1.2%)	0.090
Sarcopenia (*n*, %)	10 (2.4%)	13 (4.7%)	0.095	14 (2.9%)	3 (1.2%)	0.135
Severe sarcopenia (*n*, %)	1 (0.2%)	6 (2.2%)	0.013	4 (0.8%)	2 (0.8%)	0.944
Functional sarcopenia (*n*, %)	9 (2.1%)	16 (5.8%)	0.012	37 (7.6%)	39 (15.2%)	0.001
DM duration (years)	NC	9.5 ± 4.7		NC	8.7 ± 5.0	
Alcohol (g/week)	103.1	133.3	0.028	9.1	10.8	0.529
Smoking			0.574			0.388
Non‐smoker	108 (25.7%)	62 (22.3%)		478 (98.8%)	253 (98.4%)	
Ex‐smoker	244 (58.0%)	166 (59.7%)		4 (0.8%)	4 (1.6%)	
Current smoker	69 (16.4%)	50 (18.0%)		2 (0.4%)	0 (0.0%)	
Education			0.525			0.001
≤ 6 years	24 (5.7%)	17 (6.1%)		68 (14.1%)	60 (23.53%)	
6–12 years	263 (62.5%)	183 (66.1%)		349 (72.1%)	175 (68.6%)	
> 12 years	134 (31.8%)	77 (27.8%)		67 (13.8%)	20 (7.8%)	
Hypertension (*n*, %)	141 (33.5%)	157 (56.5%)	< 0.001	152 (31.4%)	127 (49.4%)	< 0.001
Cardiovascular disease (*n*, %)	29 (6.9%)	47 (16.9%)	< 0.001	25 (5.2%)	21 (8.2%)	0.107
Exercise			0.466			0.900
None	205 (48.7%)	135 (48.6%)		289 (59.7%)	149 (58.0%)	
Light	69 (16.4%)	37 (13.3%)		43 (8.9%)	24 (9.3%)	
Regular	147 (34.9%)	106 (38.1%)		152 (31.4%)	84 (32.7%)	
Total METs	130.3 (0–289.3)	149.6 (0–303.4)	0.260	97.7 (0–212.1)	120 (0–240.0)	0.024

Abbreviations: 2 MWT, 2‐min walking test; ASM, appendicular skeletal muscle; DM, type 2 diabetes mellitus; HDL, high‐density lipoprotein; HGS, hand grip strength (kg); HOMA‐IR, homeostasis model assessment of insulin resistance (HOMA)‐IR; IMAT, inter/intramuscular adipose tissue; IMAT index, IMAT/(TMA + IMAT) × 100; LAMA, low‐attenuation area; LAMA index, LAMA/(TMA + IMAT) × 100; METs, metabolic equivalent of task; NAMA, normal‐attenuation area; NAMA index, NAMA/(TMA + IMAT) × 100; SBP, systolic blood pressure; STST, 30‐s sit‐to‐stand test; TMA, total muscle area; VEL, usual gait speed (m/s); VEL‐F, fastest gait speed (m/s).

^a^ *p*‐value from log transformation.

### 3.2 Association Between Muscle Mass or Muscle Quality Index and Muscle Strength or Physical Performance

**FIGURE 2:**
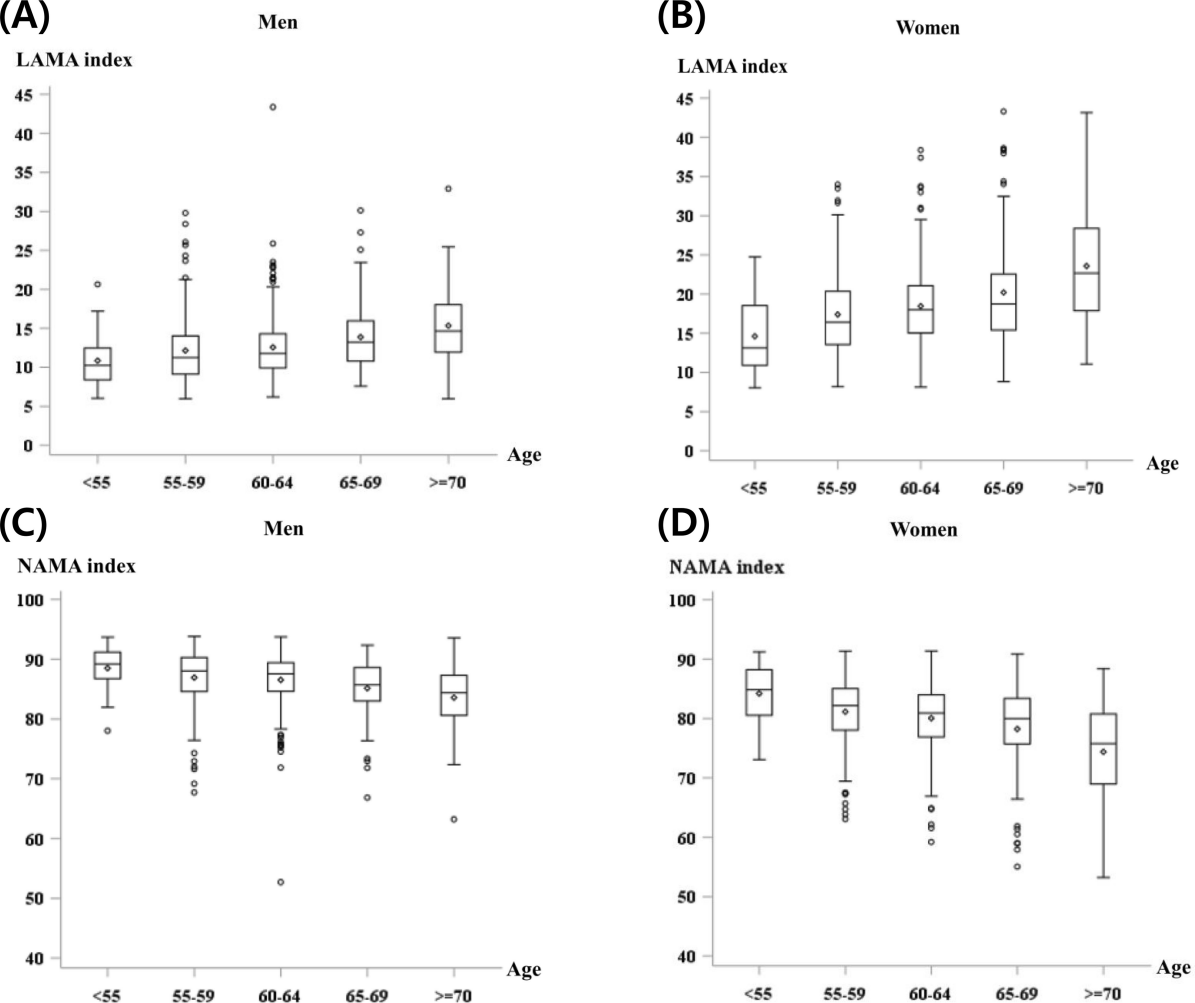
Box‐plot distribution of muscle quality index according to sex and age groups. (A) LAMA index in men, (B) LAMA index in women, (C) NAMA index in men and (D) NAMA index in women. LAMA index: LAMA/(TMA + IMAT) × 100; NAMA index: NAMA/(TMA + IMAT) × 100. IMAT, inter/intramuscular adipose tissue area; LAMA, low‐attenuation muscle area; NAMA, normal‐attenuation muscle area; TMA, total muscle area.

**FIGURE 3:**
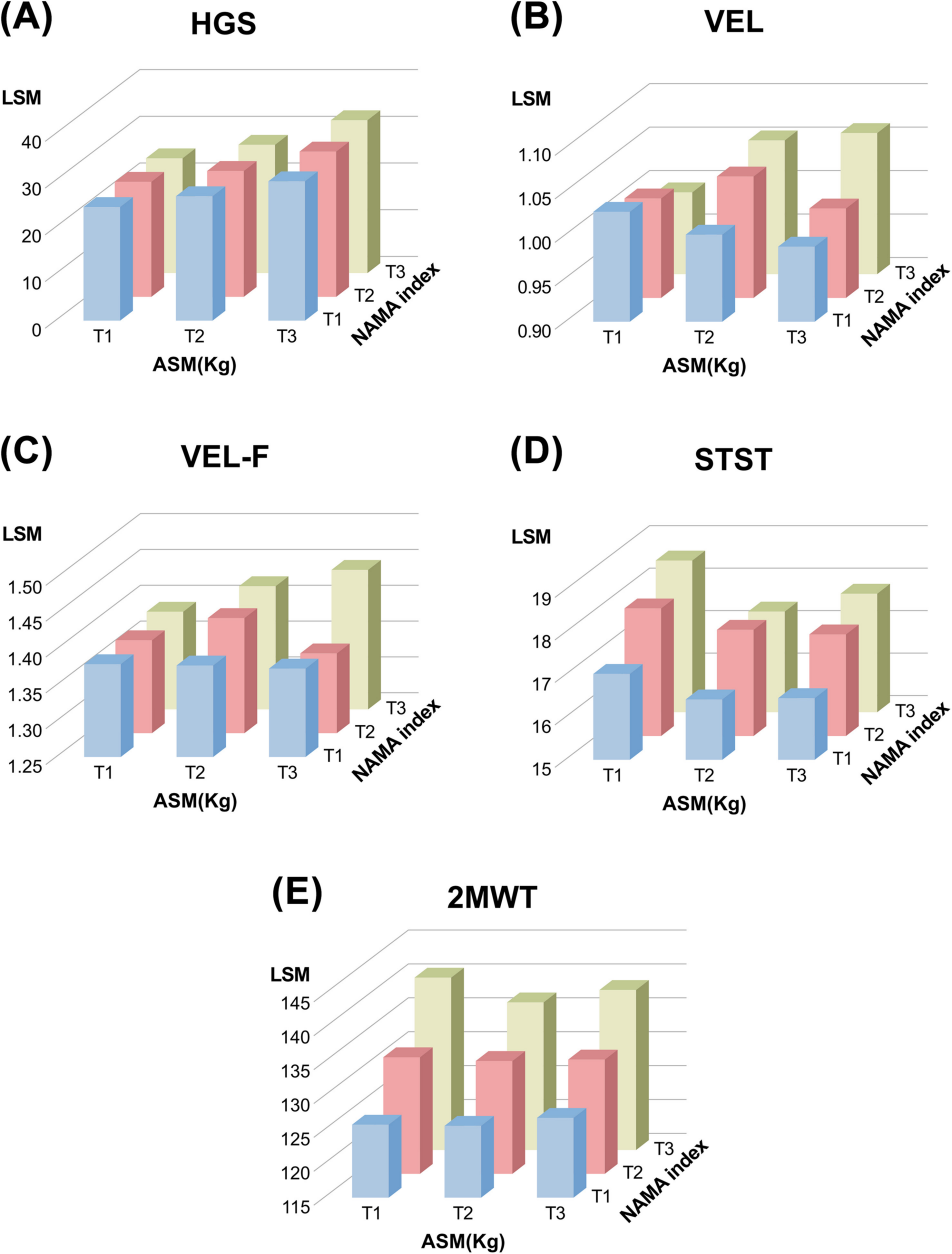
Muscle strength and physical performance according to each tertile of ASM and NAMA index adjusted by sex and age. (A) HGS, (B) VEL, (C) VEL‐F, (D) STST and (E) 2 MWT. 2 MWT; 2‐min walking test; ASM, appendicular skeletal muscle; HGS, hand grip strength; IMAT, inter/intramuscular adipose tissue area; NAMA, normal‐attenuation muscle area; NAMA index: NAMA/(TMA + IMAT) × 100; STST, 30‐s sit‐to‐stand test; T, tertiles; TMA, total muscle area; VEL, usual gait speed (m/s); VEL‐F, fastest gait speed (m/s).

With aging, the LAMA index gradually increased, and the NAMA index gradually decreased in both sexes (*p* for trend < 0.001) (Figure 2). We evaluated the association among muscle strength, physical performance and muscle mass or muscle quality index using age‐ and sex‐adjusted linear regression models (Table 2). ASM and SMI were related to HGS (*p* < 0.001); however, they were not significantly associated with VEL, VEL‐F, STST or 2MWT. TMA was positively associated with HGS, STST and 2MWT. NAMA and the NAMA index were positively associated with HGS and all physical performance tests. In contrast, LAMA and IMAT, as well as their indices, were negatively associated with physical performance tests. Regarding HGS, the LAMA and IMAT indices showed negative associations. After isolating the nine groups of participants according to the tertiles of the ASM and NAMA indices (Figure 3), the higher tertiles of the NAMA index were consistently associated with improved physical performance across all ASM tertiles. Notably, the additive effect of ASM on muscle strength was evident, demonstrating a significant trend (*p* < 0.0001).

**TABLE 2:** Sex‐ and age‐adjusted associations between muscle strength or physical performance and muscle mass or muscle quality index.

		HGS	VEL	VEL‐F	STST	2MWT
ASM (kg)	Beta	0.187	0.239	0.241	−0.024	0.000
	se	0.011	0.334	0.258	0.014	0.002
	*p*	< 0.001	0.474	0.350	0.082	0.841
SMI (ASM/height^2^)	Beta	0.039	−0.105	−0.056	−0.001	0.000
	se	0.004	0.105	0.081	0.004	0.001
	*p*	< 0.001	0.318	0.482	0.774	0.734
TMA	Beta	0.880	1.994	2.859	0.190	0.042
	se	0.079	2.289	1.765	0.093	0.015
	*p*	< 0.001	0.384	0.106	0.041	0.005
NAMA^a^	Beta	0.008	0.054	0.060	0.004	0.001
	se	0.001	0.024	0.018	0.001	0.0002
	*p*	< 0.001	0.022	0.001	< 0.001	< 0.001
LAMA^a^	Beta	0.002	−0.194	−0.144	−0.009	−0.002
	se	0.002	0.046	0.036	0.002	0.000
	*p*	0.156	< 0.001	< 0.001	< 0.001	< 0.001
IMAT^a^	Beta	−0.005	−0.354	−0.270	−0.018	−0.004
	se	0.004	0.103	0.080	0.004	0.001
	*p*	0.182	0.001	0.001	< 0.001	< 0.001
NAMA index^a^	Beta	0.001	0.048	0.041	0.002	0.001
	se	0.000	0.011	0.008	0.000	0.000
	*p*	0.010	< 0.001	< 0.001	< 0.001	< 0.001
LAMA index^a^	Beta	−0.005	−0.199	−0.162	−0.010	−0.002
	se	0.002	0.044	0.034	0.002	0.000
	*p*	0.002	< 0.001	< 0.001	< 0.001	< 0.001
IMAT index^a^	Beta	−0.010	−0.313	−0.257	−0.017	−0.004
	se	0.003	0.089	0.068	0.004	0.001
	*p*	0.001	< 0.001	< 0.001	< 0.001	< 0.001

Abbreviations: 2MWT; 2‐min walking test; ASM, appendicular skeletal muscle; HGS, hand grip strength (kg); IMAT, inter‐intramuscular adipose tissue; IMAT index, IMAT/(TMA + IMAT) × 100; LAMA, low‐attenuation area; LAMA index, LAMA/(TMA + IMAT) × 100; NAMA, normal‐attenuation area; NAMA index, NAMA/(TMA + IMAT) × 100; STST, 30‐s sit‐to‐stand test; TMA, total muscle area; VEL, usual gait speed (m/s); VEL‐F, fastest gait speed (m/s).

^a^ *p*‐value from log transformation.

### 3.3 Effect of Exercise on Muscle Quality, Strength and Physical Performance Across Diabetes Status

The relationship between the NAMA or LAMA index and muscle strength and physical performance did not differ according to diabetes status (Table 3). A subgroup analysis was conducted to evaluate the impact of exercise on the muscle quality index (Table S2). In the non‐diabetes group, regular exercise was associated with a higher NAMA index (*p* = 0.021) and a lower LAMA index (*p* = 0.006). In contrast, no significant differences were observed in the NAMA, IMAT or LAMA indices relative to exercise in the diabetes group. To evaluate the effects of exercise intensity, we stratified participants into total METs tertiles (Table S3). In the non‐diabetes group, individuals in the highest tertile of total METs demonstrated a significantly increased NAMA index (*p* = 0.010) and a decreased LAMA index (*p* = 0.002) compared to those in the lower tertiles. However, in the diabetes group, no statistically significant differences in NAMA (*p* = 0.470) or LAMA indices (*p* = 0.585) were observed across total METs tertiles.

**TABLE 3:** Linear regression analyses between muscle quality index and muscle strength or physical performance according to diabetes.

Dependent	All						Non‐diabetes						Diabetes						*p* for interaction	
	NAMA index^a^			LAMA index^a^			NAMA index^a^			LAMA index^a^			NAMA index^a^			LAMA index^a^			NAMA index^a^	LAMA index^a^
	Beta	se	*p*	Beta	se	*p*	Beta	se	*p*	Beta	se	*p*	Beta	se	*p*	Beta	se	*p*		
HGS	5.786	1.631	< 0.001	−1.644	0.402	< 0.001	4.290	2.244	0.056	−1.370	0.522	0.009	6.132	2.411	0.011	−1.565	0.643	0.015	0.778	0.709
VEL	0.254	0.064	< 0.001	−0.065	0.016	< 0.001	0.209	0.086	0.016	−0.061	0.020	0.002	0.265	0.097	0.007	−0.061	0.026	0.019	0.839	0.632
VEL‐F	0.356	0.082	< 0.001	−0.087	0.020	< 0.001	0.399	0.114	0.001	−0.100	0.027	< 0.001	0.293	0.120	0.015	−0.062	0.032	0.056	0.245	0.199
STST	7.966	1.559	< 0.001	−2.020	0.385	< 0.001	9.006	2.213	< 0.001	−2.213	0.515	< 0.001	6.215	2.208	0.005	−1.505	0.590	0.011	0.297	0.374
2 MWT	71.680	9.674	< 0.001	−17.022	2.391	< 0.001	60.113	13.122	< 0.001	−13.006	3.061	< 0.001	77.192	14.572	< 0.001	−19.878	3.895	< 0.001	0.351	0.566

*Note:* Adjusted for age, sex, appendicular skeletal muscle, exercise, alcohol consumption, smoking status, cardiovascular disease, hypertension medication, lipid‐lowering medication and education.

Abbreviations: 2 MWT; 2‐min walking test; HGS, hand grip strength (kg); IMAT, inter‐intramuscular adipose tissue; LAMA, low‐attenuation area; LAMA index, LAMA/(TMA + IMAT) × 100; NAMA, normal‐attenuation area; NAMA index, NAMA/(TMA + IMAT) × 100; STST, 30‐s sit‐to‐stand test; VEL, usual gait speed (m/s); VEL‐F, fastest gait speed (m/s).

^a^ *p*‐value from log transformation.

To account for nutritional factors, we performed an additional analysis in a subgroup of subjects with available total calorie intake data (*n* = 522). Adjusting for calorie intake did not change the relationship between NAMA and LAMA indices and muscle strength or physical performance (Table S4), nor did not alter the observed impact of exercise on NAMA and LAMA indices (Table S5).

A sensitivity analysis was conducted to confirm the robustness of the results. Excluding subjects with a prescription history of thyroid hormones or oestrogen showed similar results to those of primary analysis (Tables S6 and S7).

## 4 Discussion

In this study, muscle quality indices, including both NAMA and LAMA, correlated with muscle strength and physical performance, independent of muscle mass or diabetic status. Patients with type 2 diabetes exhibited advanced myosteatosis relative to those with normoglycaemia. Compared with participants with normoglycaemia, patients with type 2 diabetes exhibited a more pronounced decline in muscle strength and physical performance in relation to their reduction in muscle mass. Interestingly, exercise does not seem to reduce myosteatosis as effectively in individuals with diabetes as it does in individuals without diabetes. This suggests that in patients with diabetes, improving myosteatosis may require not only exercise, but also other intervention methods.

Sarcopenia, characterized by a decline in muscle mass and function, is associated with an increased risk of cardiometabolic diseases, stroke, cognitive impairment, mobility disorders and falls [1]. These conditions can lead to fractures and frailty, which contributes to increased mortality. Muscle strength or physical performance may be more critical factors for the quality of life, cardiovascular risk and mortality than muscle mass alone [26, 27, 28]. Delmonico et al. [3] demonstrated that older patients experience greater loss of muscle strength rather than muscle mass. Our study showed that age‐related changes in fat distribution led to a decrease in NAMA and an increase in LAMA and IMAT, which could induce more rapid changes in muscle strength than in muscle mass irrespective of sex. Women with diabetes had a higher muscle mass index, but lower HGS and physical performance than women without diabetes. However, they have also had higher waist circumferences, representing metabolically unhealthy body phenotype. This suggests that higher muscle mass does not necessarily correlate with improved muscle function [29]. This discrepancy can be explained more accurately by muscle quality, such as myosteatosis‐fatty infiltration in skeletal muscle [4].

Myosteatosis induces muscle atrophy, alters capillary density and reduces maximal torque production, resulting in decreased muscle function [30]. In the Health, Aging and Body Composition study involving black and white older adults aged 70–79 years demonstrated reduced muscle attenuation was associated with physical performance represented by a 6‐m walk and repeated chair stands even after adjustment for total body fat and muscle area [31]. Moreover, increased fatty acid infiltration within the thigh muscles is associated with an increased risk of hip fracture [32]. A recent study using quantitative magnetic resonance imaging (MRI) have showed that both intermuscular and intramuscular fat were negatively associated with muscle strength beyond muscle mass index in patients with type 2 diabetes. [33] Our study is the first to use CT to measure myosteatosis using various indices, such as LAMA, NAMA and IMAT, establishing its association with both muscle strength and physical performance in a large Asian population‐based cohort. Furthermore, we identified a significant association between myosteatosis and muscle function in patients with and without diabetes.

Muscle biopsy is the gold standard for the evaluation of myosteatosis. Owing to their invasiveness, radiographic tools such as CT and MRI are used to assess muscle size, composition and adipose tissue infiltration [34]. ^1^H magnetic resonance spectroscopy (MRS) has no radiation exposure and is specialized for separating intracellular and extracellular lipids but is expensive. Therefore, the MRS is primarily reserved for research purposes. Compared with MRI, CT is more accessible and affordable. CT is capable of capturing muscle size and volume as well as quantifying muscle density, expressed as muscle attenuation in HU, and facilitates the differentiation between muscle and adipose tissue, allowing the estimation of fat content within the muscle [34]. However, reference CT values remain unestablished; we defined skeletal muscle attenuation between 0 and 100 HU as TMA. Muscle regions with lower lipid content (HU 35–100) were categorized as NAMA, and muscles with higher lipid content were referred to as LAMA (HU 0–34). IMAT was quantified as −190 to −30 HU. The area HU between −29 and −1 was unmeasured. However, the area may account for ~3.5%–7% of the mid‐thigh and is associated with locations of transition around the edges of the skin and muscle fascia, which is debated for its relevance in measuring muscle area [14]. Studies that applied similar HU measurements have been previously published [13, 14].

The role of the skeletal muscle as a primary organ for glucose disposal highlights the significance of increased fat infiltration in the development of insulin resistance and metabolic diseases. Myosteatosis is linked to insulin resistance and metabolic diseases including type 2 diabetes through several mechanisms including inflammatory cytokines, decreased insulin sensitivity index and glucose disposal rate and increased cholesterol [5, 35, 36, 37]. Choe et al. [38] showed that increased fat infiltration in thigh muscles is associated with a higher risk of cardiovascular disease after adjusting for cardiometabolic risk factors. Weight loss in both diabetic and non‐diabetic individuals significantly reduces myosteatosis and enhances insulin sensitivity [7]. Exercise improves insulin resistance by promoting the translocation of glucose transporter protein type‐4 from intracellular sites to the sarcolemma and T tubules and increasing glucose uptake into the muscle [24]. Furthermore, exercise reduces fat accumulation in muscle tissues and modifies the muscle fibre composition by increasing the proportion of fast‐twitch type II fibres [39]. Consistent with these results, our study demonstrated that regular exercise was associated with less myosteatosis in non‐diabetic participants. Notably, higher METs, encompassing exercise type, intensity and frequency, significantly improved NAMA and LAMA indices. However, no significant differences due to exercise were observed in patients with diabetes. Owing to the distinctive metabolic alterations and mechanisms of muscle regeneration associated with diabetes, exercise may not reduce myosteatosis as effectively as it does in non‐diabetic individuals. Patients with type 2 diabetes are mainly characterized by type I fibre loss, which is important for energy utilization during exercise [40]. In addition, hyperglycaemia impairs muscle repair through excessive fibrosis and delayed myofiber maturation [41]. The exploration of pharmacological or nutritional interventions may be important for attenuating myosteatosis and its clinical outcomes in patients with diabetes. Nevertheless, it remains crucial to integrate appropriate resistance and high‐intensity interval training into an exercise regimen. Additionally, enhancing the dietary intake of foods rich in antioxidants and branched‐chain amino acids is essential for supporting muscle health and facilitating recovery [40]. Our study did not conduct a detailed analysis of the types of exercises performed. Further studies are needed to explore the influence of different types of exercise on myosteatosis, potentially offering insights into personalized physical activity recommendations for individuals with diabetes.

This study had several limitations. First, a cross‐sectional design was employed. We could not evaluate the causal relationship between muscle quality, muscle strength, and physical performance. Second, we used only the thigh muscles to calculate muscle attenuation although the CT density of the cross‐sectional area of the muscle and adipose tissue from the lower extremities was highly correlated to that of the whole body in previous studies [5]. In addition, we used a single threshold (34 HU) to differentiate between normal and low‐attenuation muscle areas, which may not account for variations across different demographics. Future research should incorporate multiple measurement sites, explore age‐ and gender‐specific thresholds and validate findings against other assessment methods such as MRI or muscle biopsies. Third, our study was conducted in relatively healthy volunteers who could undergo all physical examinations. Therefore, the generalizability of our results to the entire elderly population may be limited. However, this study leveraged a large cohort of elderly Koreans, including a significant number of individuals with diabetes, thus offering a unique opportunity to conduct a comparative analysis across individuals with diabetes and those without. This study advances the understanding of the effects of myosteatosis on muscle strength and physical performance. Furthermore, professionally trained research staff measured the myosteatosis area, employed highly accurate and reproducible automated measurement techniques and performed physical examinations for physical function according to standardized study protocols.

In this population‐based cohort study, we demonstrated that lesser myosteatosis in the thigh is associated with greater muscle strength and better physical performance in both men and women, regardless of muscle mass or diabetes status. Participants with diabetes had a more pronounced decrease in physical performance and muscle strength than muscle mass, accompanied by greater myosteatosis. Future prospective studies are needed to demonstrate whether reducing myosteatosis prevents sarcopenia, as well as its practical applications in clinical settings.

## Conflicts of Interest

The authors declare no conflicts of interest.

## Supporting information


**Table S1** Age‐adjusted mean differences of muscle mass and physical performance by sex and diabetes.
**Table S2** Subgroup analysis according to regular exercise.
**Table S3** Subgroup analysis according to METs.
**Table S4** Linear regression analyses between muscle quality index and muscle strength or physical performance according to diabetes after adjustment for total calories.
**Table S5** Subgroup analysis according to regular exercise after adjustment for total caloric intake.
**Table S6** Sensitivity analysis: linear regression analyses between muscle quality index and muscle strength or physical performance according to diabetes, excluding subjects taking thyroid medication and oestrogen.
**Table S7** Sensitivity analysis: subgroup analysis according to regular exercise after excluding subjects taking thyroid medication and oestrogen.
