# Supplementary material for: Impact of Muscle Quality on Muscle Strength and Physical Performance Beyond Muscle Mass or Diabetes Status
Source: J Cachexia Sarcopenia Muscle. 2025 Mar 4;16(2):e13760. doi: 10.1002/jcsm.13760 (PMC11876848; doi:10.1002/jcsm.13760)
Supplement: Supplementary file 1 — Table S1 Age‐adjusted mean differences of muscle mass and physical performance by sex and diabetes. Table S2 Subgroup analysis according to regular exercise. Table S3 Subgroup analysis according to METs. Table S4 Linear regression analyses between muscle quality index and muscle strength or physical performance according to diabetes after adjustment for total calories. Table S5 Subgroup analysis according to regular exercise after adjustment for total caloric intake. Table S6 Sensitivity analysis: linear regression analyses between muscle quality index and muscle strength or physical performance according to diabetes, excluding subjects taking thyroid medication and oestrogen. Table S7 Sensitivity analysis: subgroup analysis according to regular exercise after excluding subjects taking thyroid medication and oestrogen. [file JCSM-16-e13760-s001.docx]

Supplementary Table 1. Age-adjusted mean differences of muscle mass and physical performance by sex and diabetes

|  | Men | | | |  | Women | | | |  |
| --- | --- | --- | --- | --- | --- | --- | --- | --- | --- | --- |
|  | Non-diabetes | | Diabetes | |  | Non-diabetes | | Diabetes | |  |
|  | lsmean | se | lsmean | se | P-value | lsmean | se | lsmean | se | P-value |
| ASM (kg) | 22.64 | 0.13 | 22.41 | 0.15 | 0.264 | 15.29 | 0.08 | 15.67 | 0.11 | 0.006 |
| ASM/height^2^ | 8.05 | 0.04 | 8.03 | 0.04 | 0.767 | 6.34 | 0.03 | 6.53 | 0.04 | <0.001 |
| TMA (cm^3^) | 135.86 | 0.85 | 134.32 | 1.04 | 0.255 | 91.48 | 0.56 | 94.39 | 0.77 | 0.003 |
| NAMA (cm^3^)* | 117.4 | 115.77 - 119.04 | 114.08 | 112.14 - 116.06 | 0.002 | 73.18 | 72.12 - 74.26 | 74.79 | 73.30 - 76.32 | 0.089 |
| NAMA index* | 86.56 | 86.10 - 87.02 | 84.80 | 84.24 - 85.36 | <0.001 | 79.55 | 78.97 - 80.14 | 78.67 | 77.88 - 79.48 | 0.085 |
| LAMA (cm^3^)* | 16.25 | 15.77 - 16.74 | 17.79 | 17.15 - 18.45 | <0.001 | 16.46 | 16.02 - 16.9 | 17.36 | 16.73 - 18.02 | 0.023 |
| LAMA index* | 11.98 | 11.65 - 12.32 | 13.22 | 12.78 - 13.69 | <0.001 | 17.89 | 17.43 - 18.36 | 18.26 | 17.63 - 18.92 | 0.357 |
| IMAT* | 0.89 | 0.83- 0.95 | 1.14 | 1.05 – 1.24 | <0.001 | 1.11 | 1.05 – 1.18 | 1.33 | 1.23 – 1.43 | <0.001 |
| IMAT index * | 0.68 | 0.64 - 0.72 | 0.88 | 0.81 - 0.95 | <0.001 | 1.23 | 1.17 - 1.30 | 1.42 | 1.31 - 1.53 | 0.004 |
| Handgrip strength, HGS (kg) | 35.95 | 0.27 | 34.44 | 0.33 | 0.001 | 21.25 | 0.18 | 21.05 | 0.25 | 0.533 |
| Usual gait speed, VEL (m/s) | 1.11 | 0.01 | 1.07 | 0.01 | 0.005 | 1.04 | 0.01 | 1.04 | 0.01 | 0.855 |
| Fastest gait speed, VEL-F (m/s) | 1.51 | 0.01 | 1.46 | 0.01 | 0.003 | 1.38 | 0.01 | 1.38 | 0.01 | 0.979 |
| 30-second sit-to-stand test, STST | 19.07 | 0.22 | 18.45 | 0.27 | 0.0801 | 17.00 | 0.18 | 16.44 | 0.25 | 0.070 |
| 2-minute walking test, 2MWT | 149.52 | 1.35 | 137.20 | 1.66 | <0.001 | 132.51 | 1.16 | 130.53 | 1.60 | 0.321 |

* P value from log transformation

lsmean: least squares means; se: standard error; ASM: appendicular skeletal muscle; TMA: total muscle area; NAMA: normal-attenuation area; LAMA: low-attenuation area; IMAT: inter-intramuscular adipose tissue; NAMA index: NAMA/(TMA + IMAT)X 100; LAMA index: LAMA/ (TMA + IMAT)X 100; IMAT index, IMAT/(TMA+IMAT) x 100

Supplementary Table 2. Subgroup analysis according to regular exercise

|  | Non-diabetes | | | | Diabetes | | | |  |  | post-hoc p value | | | | | |
| --- | --- | --- | --- | --- | --- | --- | --- | --- | --- | --- | --- | --- | --- | --- | --- | --- |
| Exercise | no (1) | | yes (2) | | no (3) | | yes (4) | | overall | p |  |  |  |  |  |  |
|  | lsmean | se | lsmean | se | lsmean | se | lsmean | se | p value | interaction | 1 vs 2 | 1 vs 3 | 1 vs 4 | 2 vs 3 | 2 vs 4 | 3 vs 4 |
| NAMAindex* | 82.47 | 81.56-83.38 | 83.67 | 82.62-84.73 | 81.64 | 80.73-82.57 | 81.82 | 80.73-82.92 | <0.001 | 0.129 | **0.021** | 0.183 | 0.563 | <0.001 | 0.005 | **0.987** |
| IMATindex* | 0.99 | 0.89-1.09 | 0.92 | 0.82-1.03 | 1.15 | 1.04-1.27 | 1.13 | 1-1.27 | <0.001 | 0.487 | 0.404 | 0.003 | 0.065 | <0.001 | 0.004 | 0.987 |
| LAMAindex* | 14.99 | 14.34-15.67 | 14.01 | 13.32-14.75 | 15.40 | 14.71-16.12 | 15.52 | 14.7-16.39 | <0.001 | **0.023** | **0.006** | 0.546 | 0.489 | <0.001 | 0.001 | **0.990** |

*: IQR, p-value from Log transformation

lsmean: least squares means; se: standard error; ASM: appendicular skeletal muscle; TMA: total muscle area; NAMA: normal-attenuation area; LAMA: low-attenuation area; IMAT: inter-intramuscular adipose tissue; NAMA index: NAMA/(TMA + IMAT)X 100; LAMA index: LAMA/ (TMA + IMAT)X 100; IMAT index, IMAT/(TMA+IMAT) x 100

Adjusted for age, sex, appendicular skeletal muscle, alcohol consumption, smoking status, cardiovascular disease, hypertension medication, lipid-lowering medication, and education.

Supplementary Table 3. Subgroup analysis according to METs

|  | Non-diabetes | | | | Diabetes | | | |  |  | post-hoc p value | |
| --- | --- | --- | --- | --- | --- | --- | --- | --- | --- | --- | --- | --- |
| Total METs | T1&T2 (1) | | T3 (2) | | T1&T2 (3) | | T3 (4) | | overall | p |  |  |
|  | lsmean | se | lsmean | se | lsmean | se | lsmean | se | p value | interaction | 1vs2 | 3vs4 |
| N | 614 |  | 291 |  | 337 |  | 198 |  |  |  |  |  |
| Total METs | 53.46 | 63.95 | 416.75 | 230.13 | 61.82 | 67.08 | 405.63 | 234.97 |  |  | <.001 | <.001 |
| NAMAindex* | 82.44 | 81.54-83.35 | 83.76 | 82.7-84.82 | 81.44 | 80.53-82.37 | 82.20 | 81.12-83.30 | <0.001 | 0.417 | **0.010** | **0.470** |
| IMATindex* | 0.98 | 0.89-1.08 | 0.93 | 0.83-1.04 | 1.18 | 1.07-1.31 | 1.07 | 0.95-1.20 | <0.001 | 0.478 | 0.654 | 0.260 |
| LAMAindex* | 15.01 | 14.35-15.69 | 13.95 | 13.25-14.68 | 15.61 | 14.91-16.35 | 15.11 | 14.32-15.94 | <0.001 | 0.216 | **0.002** | **0.585** |

*: IQR, p-value from Log transformation

METs; metabolic equivalent of task, lsmean: least squares means; se: standard error; ASM: appendicular skeletal muscle; TMA: total muscle area; NAMA: normal-attenuation area; LAMA: low-attenuation area; IMAT: inter-intramuscular adipose tissue; NAMA index: NAMA/(TMA + IMAT)X 100; LAMA index: LAMA/ (TMA + IMAT)X 100; IMAT index, IMAT/(TMA+IMAT) x 100

Adjusted for age, sex, appendicular skeletal muscle, alcohol consumption, smoking status, cardiovascular disease, hypertension medication, lipid-lowering medication, and education.

Supplementary Table 4. Linear regression analyses between muscle quality index and muscle strength or physical performance according to diabetes after adjustment for total calories

|  | **ALL** | | | | | | **Non-diabetes** | | | | | | **Diabetes** | | | | | | **p for interaction** | |
| --- | --- | --- | --- | --- | --- | --- | --- | --- | --- | --- | --- | --- | --- | --- | --- | --- | --- | --- | --- | --- |
|  | NAMAindex* | | | LAMAindex* | | | NAMAindex* | | | LAMAindex* | | | NAMAindex* | | | LAMAindex* | | | NAMA  index* | LAMAindex* |
| **Dependent** | beta | se | p | beta | se | p | beta | se | p | beta | se | p | beta | se | p | beta | se | p |  |  |
| **HGS** | 5.83 | 3.00 | 0.052 | -1.83 | 0.71 | 0.010 | 8.29 | 4.27 | 0.053 | -2.32 | 0.97 | 0.018 | 1.58 | 4.19 | 0.706 | -0.69 | 1.05 | 0.510 | 0.236 | 0.275 |
| **VEL** | 0.36 | 0.13 | 0.005 | -0.11 | 0.03 | <.001 | 0.32 | 0.18 | 0.075 | -0.10 | 0.04 | 0.017 | 0.39 | 0.18 | 0.031 | -0.12 | 0.04 | 0.006 | 0.388 | 0.489 |
| **VEL-F** | 0.42 | 0.16 | 0.011 | -0.13 | 0.04 | 0.001 | 0.56 | 0.23 | 0.018 | -0.16 | 0.05 | 0.004 | 0.27 | 0.23 | 0.239 | -0.09 | 0.06 | 0.120 | 0.101 | 0.118 |
| **STST** | 8.44 | 2.94 | 0.004 | -2.11 | 0.70 | 0.003 | 7.40 | 4.07 | 0.070 | -2.10 | 0.93 | 0.025 | 10.48 | 4.35 | 0.017 | -2.34 | 1.09 | 0.034 | 0.650 | 0.795 |
| **2 MWT** | 98.02 | 17.20 | <.001 | -23.54 | 4.09 | <.001 | 87.57 | 23.75 | <.001 | -19.83 | 5.43 | <.001 | 106.96 | 24.84 | <.001 | -26.95 | 6.20 | <.001 | 0.193 | 0.300 |

*: IQR, p-value from Log transformation

NAMA, normal-attenuation area; LAMA, low-attenuation area; IMAT, inter-intramuscular adipose tissue; NAMA index, NAMA/ (TMA + IMAT) ×100; LAMA index, LAMA / (TMA + IMAT) × 100; HGS, hand grip strength (kg); VEL, usual gait speed (m/s); VEL-F, fastest gait speed (m/s); STST, 30-second sit-to-stand test; 2 MWT; 2-minute walking test

Adjusted for age, sex, exercise, appendicular skeletal muscle, alcohol consumption, smoking status, cardiovascular disease, hypertension medication, lipid-lowering medication, education, and total calories (Subjects number: 522).

Supplementary table 5. Subgroup analysis according to regular exercise after adjustment for total caloric intake

|  | Non-diabetes | | | | Diabetes | | | |  |  | post-hoc p value | |
| --- | --- | --- | --- | --- | --- | --- | --- | --- | --- | --- | --- | --- |
| Exercise | no (1) | | yes (2) | | no (3) | | yes (4) | | overall | p |  |  |
|  | lsmean | se | lsmean | se | lsmean | se | lsmean | se | p value | interaction | 1 vs 2 | 3 vs 4 |
| NAMAindex* | 81.28 | 79.95-82.64 | 82.67 | 81.08-84.28 | 81.05 | 79.69-82.43 | 81.21 | 79.71-82.75 | 0.102 | 0.228 | 0.135 | 0.997 |
| IMATindex* | 1.18 | 1.01-1.37 | 1.11 | 0.93-1.33 | 1.34 | 1.15-1.56 | 1.33 | 1.12-1.58 | 0.077 | 0.667 | 0.861 | 0.9998 |
| LAMAindex* | 16.10 | 15.02-17.26 | 14.94 | 13.77-16.21 | 15.91 | 14.82-17.08 | 16.16 | 14.93-17.48 | 0.121 | 0.082 | 0.104 | 0.980 |

*: IQR, p-value from Log transformation

lsmean: least squares means; se: standard error; ASM: appendicular skeletal muscle; TMA: total muscle area; NAMA: normal-attenuation area; LAMA: low-attenuation area; IMAT: inter-intramuscular adipose tissue; NAMA index: NAMA/(TMA + IMAT)X 100; LAMA index: LAMA/ (TMA + IMAT)X 100; IMAT index, IMAT/(TMA+IMAT) x 100

Adjusted for age, sex, appendicular skeletal muscle, alcohol consumption, smoking status, cardiovascular disease, hypertension medication, lipid-lowering medication, education, and total calories (Subjects’ number: 522).

Supplementary table 6. Sensitivity analysis: Linear regression analyses between muscle quality index and muscle strength or physical performance according to diabetes, excluding subjects taking thyroid medication and estrogen

|  | **ALL** | | | | | | **Non-diabetes** | | | | | | **Diabetes** | | | | | | **p for interaction** | |
| --- | --- | --- | --- | --- | --- | --- | --- | --- | --- | --- | --- | --- | --- | --- | --- | --- | --- | --- | --- | --- |
|  | NAMAindex* | | | LAMAindex* | | | NAMAindex* | | | LAMAindex* | | | NAMAindex* | | | LAMAindex* | | | NAMAindex* | LAMAindex* |
| **Dependent** | beta | se | p | beta | se | p | beta | se | p | beta | se | p | beta | se | p | beta | se | p |  |  |
| **HGS** | 5.26 | 1.692 | 0.002 | -1.53 | 0.42 | <.001 | 3.77 | 2.33 | 0.106 | -1.27 | 0.54 | 0.018 | 5.56 | 2.51 | 0.027 | -1.39 | 0.67 | 0.039 | 0.752 | 0.569 |
| **VEL** | 0.29 | 0.07 | <.001 | -0.07 | 0.02 | <.001 | 0.25 | 0.09 | 0.005 | -0.07 | 0.02 | 0.001 | 0.29 | 0.10 | 0.004 | -0.07 | 0.03 | 0.009 | 0.542 | 0.421 |
| **VEL-F** | 0.38 | 0.09 | <.001 | -0.09 | 0.02 | <.001 | 0.42 | 0.12 | <.001 | -0.11 | 0.03 | <.001 | 0.32 | 0.13 | 0.011 | -0.07 | 0.03 | 0.044 | 0.188 | 0.135 |
| **STST** | 8.39 | 1.62 | <.001 | -2.09 | 0.40 | <.001 | 9.88 | 2.31 | <.001 | -2.36 | 0.53 | <.001 | 6.07 | 2.30 | 0.009 | -1.45 | 0.62 | 0.019 | 0.238 | 0.353 |
| **2MWT** | 72.61 | 10.06 | <.001 | -17.32 | 2.48 | <.001 | 62.24 | 13.71 | <.001 | -13.42 | 3.19 | <.001 | 75.56 | 15.04 | <.001 | -19.75 | 4.03 | <.001 | 0.215 | 0.473 |

*: IQR, p-value from Log transformation

NAMA, normal-attenuation area; LAMA, low-attenuation area; IMAT, inter-intramuscular adipose tissue; NAMA index, NAMA/ (TMA + IMAT) ×100; LAMA index, LAMA / (TMA + IMAT) × 100; HGS, hand grip strength(kg); VEL, usual gait speed (m/s); VEL-F, fastest gait speed (m/s); STST, 30-second sit-to-stand test; 2 MWT; 2-minute walking test

Adjusted for age, sex, exercise, appendicular skeletal muscle, alcohol consumption, smoking status, cardiovascular disease, hypertension medication, lipid-lowering medication, and education. (Subjects’ number: 1375)

Supplementary table 7. Sensitivity analysis: Subgroup analysis according to regular exercise after excluding subjects taking thyroid medication and estrogen

|  | Non-diabetes | | | | Diabetes | | | |  |  | post-hoc p value | |
| --- | --- | --- | --- | --- | --- | --- | --- | --- | --- | --- | --- | --- |
| Exercise | no (1) | | yes (2) | | no (3) | | yes (4) | | overall | p |  |  |
|  | lsmean | se | lsmean | se | lsmean | se | lsmean | se | p value | interaction | 1 vs 2 | 3 vs 4 |
| NAMAindex* | 82.35 | 81.44-83.27 | 83.47 | 82.42-84.54 | 81.53 | 80.61-82.46 | 81.75 | 80.64-82.87 | <.001 | 0.187 | 0.040 | 0.977 |
| IMATindex* | 1.00 | 0.90-1.10 | 0.94 | 0.84-1.06 | 1.17 | 1.06-1.29 | 1.15 | 1.02-1.30 | <.001 | 0.625 | 0.621 | 0.99 |
| LAMAindex* | 15.09 | 14.42-15.78 | 14.17 | 13.46-14.92 | 15.50 | 14.80-16.23 | 15.64 | 14.80-16.52 | <.001 | 0.033 | 0.013 | 0.987 |

*: IQR, p-value from Log transformation

lsmean: least squares means; se: standard error; ASM: appendicular skeletal muscle; TMA: total muscle area; NAMA: normal-attenuation area; LAMA: low-attenuation area; IMAT: inter-intramuscular adipose tissue; NAMA index: NAMA/(TMA + IMAT)X 100; LAMA index: LAMA/ (TMA + IMAT)X 100

Adjusted for age, sex, appendicular skeletal muscle, alcohol consumption, smoking status, cardiovascular disease, hypertension medication, lipid-lowering medication, and education. (Subjects’ number: 1375)
